# Berberrubine attenuates mucosal lesions and inflammation in dextran sodium sulfate-induced colitis in mice

**DOI:** 10.1371/journal.pone.0194069

**Published:** 2018-03-14

**Authors:** Xiu-Ting Yu, Yi-Fei Xu, Yan-Feng Huang, Chang Qu, Lie-Qiang Xu, Zi-Ren Su, Hui-Fang Zeng, Lin Zheng, Tie-Gang Yi, Hui-Lin Li, Jian-Ping Chen, Xiao-Jun Zhang

**Affiliations:** 1 The First Affiliated Hospital of Chinese Medicine, Guangzhou University of Chinese Medicine, Guangzhou, Guangdong, PR China; 2 School of Chinese Materia Medica, Guangzhou University of Chinese Medicine, Guangzhou, Guangdong, PR China; 3 Guangdong Provincial Key Laboratory of New Chinese Medicinal Development and Research, Mathematical Engineering Academy of Chinese Medicine, Guangzhou University of Chinese Medicine, Guangzhou, Guangdong, PR China; 4 Dongguan Mathematical Engineering Academy of Chinese Medicine, Guangzhou University of Chinese Medicine, Dongguan, Guangdong, PR China; 5 Shenzhen Key Laboratory of Hospital Chinese Medicine Preparation, Shenzhen Traditional Chinese Medicine Hospital, Guangzhou University of Chinese Medicine, Shenzhen, PR China; Toho Daigaku, JAPAN

## Abstract

Ulcerative colitis (UC) is a chronic relapsing disease without satisfactory treatments, in which intestinal inflammation and disrupted intestinal epithelial barrier are two main pathogeneses triggering UC. Berberrubine (BB) is deemed as one of the major active metabolite of berberine (BBR), a naturally-occurring isoquinoline alkaloid with appreciable anti-UC effect. This study aimed to comparatively investigate the therapeutic effects of BB and BBR on dextran sodium sulfate (DSS)-induced mouse colitis model, and explore the potential underlying mechanism. Results revealed that BB (20 mg/kg) produced a comparable therapeutic effect as BBR (50 mg/kg) and positive control sulfasalazine (200 mg/kg) by significantly reducing the disease activity index (DAI) with prolonged colon length and increased bodyweight as compared with the DSS group. BB treatment was shown to significantly ameliorate the DSS-induced colonic pathological alternations and decreased histological scores. In addition, BB markedly attenuated colonic inflammation by alleviating inflammatory cell infiltration and inhibiting myeloperoxidase (MPO) and cytokines (TNF-α, IFN-γ, IL-1β, IL-6, IL-4 and IL-10) productions in DSS mice. Furthermore, BB treatment substantially upregulated the expression of tight junction (TJ) proteins (zonula occludens-1, zonula occludens-2, claudin-1, occludin) and mRNA expression of mucins (mucin-1 and mucin-2), and decreased the Bax/Bcl-2 ratio. In summary, BB exerted similar effect to its analogue BBR and positive control in attenuating DSS-induced UC with much lower dosage and similar mechanism. The protective effect observed may be intimately associated with maintaining the integrity of the intestinal mucosal barrier and mitigating intestinal inflammation, which were mediated at least partially, via favorable modulation of TJ proteins and mucins and inhibition of inflammatory mediators productions in the colonic tissue. This is the first report to demonstrate that BB possesses pronounced anti-UC effect similar to BBR and sulfasalazine with much smaller dosage. BB might have the potential to be further developed into a promising therapeutic option in the treatment of UC.

## Introduction

Ulcerative colitis (UC), a subtype of inflammatory bowel disease (IBD), is clinically characterized by acute abdominal pain, weight loss, diarrhea, even hematochezia which severely lower the quality of life [1]. Nowadays, there are different drugs for UC treatment, including 5-aminosalicylic acid, steroid hormone, immunosuppressive agents and anti-tumor necrosis factor-α (anti-TNF-α) agent. However, the frequency and severity of side effects, inconvenient dosing regimen, and partially prohibitive price limit their clinical application [2]. Therefore, it is of great significance to seek effective alternative for the treatment of UC.

Inflammation responses are one of the most crucial factors causing UC [3]. The increased pro-inflammatory cytokines such as TNF-α, IFN-γ, and IL-1β, extend the inflammatory cascade and eventually lead to intestinal/colonic tissue damage in UC induced by DSS [4]. Moreover, the onset of UC is accompanied by obvious diffused intestinal inflammation which is closely associated with the increased permeability of intestinal epithelial barrier [5, 6].

The intestinal epithelial barrier, physically protecting the intestine from luminal bacteria and toxins, is composed of the mucous layer, epithelial cells and intercellular junctions. Components of the mucous layer are mucins, including *mucin-1* and *mucin-2*, which are secreted by intestinal epithelium goblet cells and markedly decreased in colitis subjects [5, 7]. Tight junctions (TJs) are the most apical structure of the junction complex, which is intercellular structure in epithelial cells and play crucial roles in cell-cell recognition and paracellular motion of substances. TJs regulate the mucosal barrier capability [8], which comprised of various transmembrane proteins including occluding and claudins, coupling each other with zonaoccludens (ZO, cytoplasmic peripheral membrane proteins including ZO-1 and ZO-2) within cells [9, 10]. Once the barrier is impaired, bacteria and toxins may easily penetrate and aggravate the release of multiple cytokines, thus evoking the occurrence and development of UC. Therefore, maintaining the integrity of the gut barrier structure and function provide an invaluable contribution to the treatment of UC, and strategies based on this are now essential lines of treatment for UC [11].

Traditional Chinese medicines (TCMs) remain a fundamental therapeutic solution in the treatment of various diseases due to their long history of clinical practice and reliable therapeutic efficacy. Rhizoma Coptidis (the rhizoma of *Coptischinensis* Franch., Huanglian in Chinese), officially listed in the *Chinese Pharmacopoeia*, has been used in China for a long history to treat gastroenteritis including abdominal pain, diarrhea and IBD [12]. Berberine (BBR), the most abundant and major active isoquinoline alkaloid of Rhizoma Coptidis [13], has been reported to exert therapeutic effect on colitis and inhibits inflammatory responses in colonic macrophages and epithelial cells in DSS-treated mice [14]. Indeed, BBR is a safe and effective agent commonly used for the treatment of gastrointestinal disorders like diarrhea in China. However, there are some limitations of BBR due to its low bioavailability and poor intestinal absorption [15]. BBR undergoes extensive metabolism *in vivo* after oral administration which results in its extremely low plasma exposure [16]. Hence, increasing researchers have focused their attention on the metabolites of BBR, which were also believed to contribute a lot to its pharmacological effects [17].

**Fig 1:**
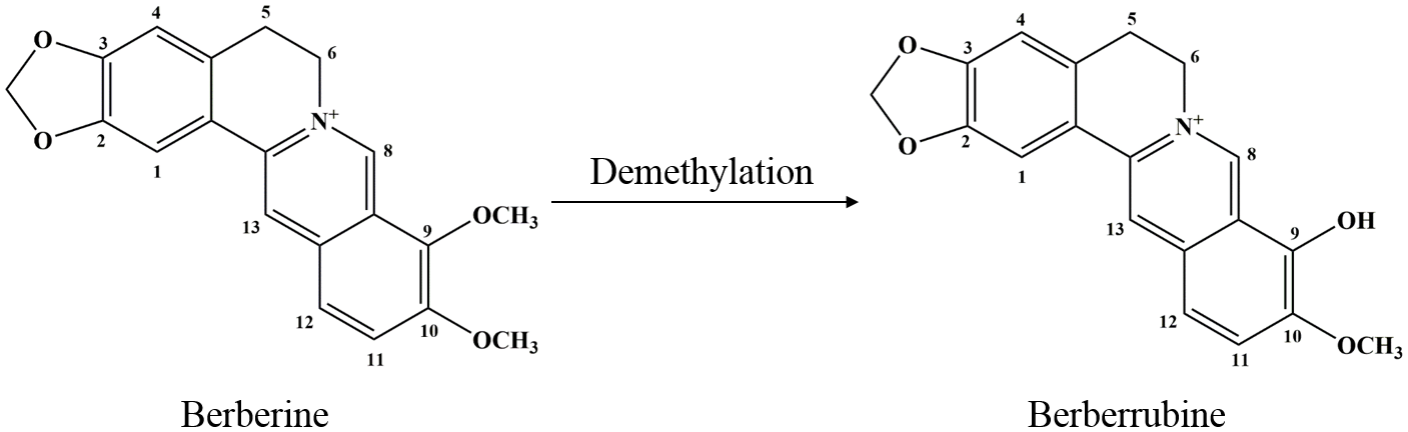
Chemical structures of berberine and berberrubine.

Berberrubine (BB), one of the main metabolites of BBR *in vivo* [18] (Fig 1), is more lipophilic than BBR and has higher plasma concentration after BBR oral administration owing to its more efficient intestinal absorption. Previous study has suggested that it is potentially more pharmacologically active than BBR [19]. Indeed, BBR and BB are both found in the medicinal plant *Berberis vulgaris*, which has been shown to possess anti-UC effect [20, 21]. Increasing evidences have shown that BB exerted multiple activities, such as anti-inflammation [13], anti-microbial [22], anti-tumor [23], lipid-lowering [24], and anti-oxidation [25]. In this study, we made pioneering endeavor to assess the potential protective effects and underlying mechanism of BB in comparison with BBR in DSS-induced colitis by measuring macroscopic score, histological alternations, inflammation markers such as MPO and pro-inflammatory cytokines productions. In addition, we further detected the expression of colonic TJs and apoptosis-related proteins, and the mRNA expression of colonic mucins. Results indicated that BB effectively prevented DSS-induced colitis in mice, and the protective effect might be intimately associated with attenuating intestinal inflammation and maintaining the integrity of the intestinal mucosal barrier.

## Materials and methods

### Chemicals and reagents

Berberine hydrochloride (purity > 98% by HPLC) was provided by Xi’an Realin Biotechnology Co. Ltd. (Xi’an, China). Dextran sulfate sodium (DSS) was obtained from MP Biomedicals (LLC, France). Sulfasalazine (SASP) was supplied by Sunve Pharmaceutical Co. Ltd. (Shanghai, China). MPO detection kit was purchased from Nanjing Jiancheng Bioenginerring Institute (Nanjing, China). ELISA kits (TNF-α, IFN-γ, IL-6, IL-1β, IL-4 and IL-10) were purchased from eBioscience (MA, USA). BCA protein assay kit was purchased from Thermo Scientific Pierce (Rockford, USA). Sodium carboxymethyl cellulose (CMC-Na) was purchased from Guangzhou Chemical Reagent Factory (Guangzhou, China). All other reagents and chemicals used in the study were of analytical grade.

### Synthesis of berberrubine

Berberrubine was synthesized according to the previous study with some modifications [26]. Briefly, berberine hydrochloride (3 g) was microwaved at 180 °C for 30 min under vacuum condition, then dissolved in deionized water (300 ml), extracted by chloroform extraction (300 ml once for 3 times) and dried using the rotary evaporation apparatus to get the crude product, which was purified and concentrated using silica gel column chromatography (CH_2_Cl_2_: MeOH = 10:1, v/v) to obtain the red compound (berberrubine). Its structural identity was confirmed by comparing its spectral data (MS, 1H- and 13C-NMR) with those published previously [27]. Its purity was determined to exceed 95.58% by HPLC analysis.

### Experimental design and sample collection

Six-week-old male Balb/c mice were obtained from the Laboratory Animal Center of Guangzhou University of Chinese Medicine (Guangzhou, China). Mice were housed under standard conditions at controlled temperature (22 ± 2 °C), humidity (50 ± 10%), and light (12 h light/dark cycle) and fed with standard diets and tap water *ad libitum*. Animal experiments were approved by the Institutional Animal Care and Welfare Committee of Guangzhou University of Chinese Medicine and performed in accordance with institutional protocols (2016047).

After acclimatization for 7 days, mice were divided into 6 groups based on the average body weight (n = 12 per group), control group, DSS group, BBL (berberrubine, 10 mg/kg) group, BBH (berberrubine, 20 mg/kg) group, BBR (berberine, 50 mg/kg, control drug) and sulfasalazine (SASP, 200 mg/kg, positive drug). BB, BBR and SASP were also dissolved in 0.5% CMC-Na. Except the control group, colitis was induced by oral intake of 3% DSS (w/v, dissolved in drinking water) for 7 days in the other 5 groups. During the experimental period, normal control was given distilled water and DSS group was given 0.5% CMC-Na by gavage. Other groups were orally administered with the corresponding tested articles at the doses mentioned above (0.1 ml/10 g body weight) once daily for 7 consecutive days. The dosage of BBR was selected based on Lee’s study [28], and the dose of BB was adopted according to previous investigation [23] and our preliminary experiment.

Animals were euthanized by an overdose of inhalational diethyl ether on day 8 and colons were collected. The colon length, an indicator of colitis, was gauged from proximal rectum to the ileocecal junction [3]. Whereafter, colon was divided into 4 different pieces. One piece was placed in 4% paraformaldehyde for histological observation. The other piece was homogenized for MPO and cytokines measurements. The remaining two parts were stored at −80°C for further Western blotting and RT-PCR analyses. The colonic samples are all taken from the same position in the colons for every application.

### Assessment of disease activity

During the experiment, body weight, stool character, and fecal occult blood were recorded daily. The disease activity index (DAI) was calculated by scoring weight loss, stool character and fecal occult blood based on the scoring system as shown in Table 1 as previously described [29].

**Table 1:** Scoring of disease activity index (DAI).

Score	Bodyweight loss	Stool character	Fecal occult blood
0	0	Normal formed	Negative
1	= 1–5%		
2	= 5–10%	Loose stool	Positive
3	= 10–20%		
4	>20%	Diarrhea	Gross bleeding

### Histopathological examination

After 4% paraformaldehyde fixation for 24 h, colon tissues were then dehydrated, embedded in paraffin, sliced to 5 μm-thickness sections, and stained with H&E following routine protocol. Finally, slides were observed under a microscope. For each sample, six horizons were selected to assess the colonic inflammation and lesions degree, which were denoted as histological scores, according to the previous criterion [30].

### Measurement of MPO activity and cytokines concentrations

The activity of MPO in the colonic tissue was measured using a MPO detection kit and the levels of cytokines (TNF-α, IFN-γ, IL-6, IL-1β, IL-4 and IL-10) were quantified by ELISA according to the instruction of manufacturers. To determine the MPO activity and the levels of these cytokines, tissues were homogenized with lysis buffer for 1 min and centrifuged at 14000 g, 4 °C for 15 min. Supernatants were collected for subsequent measurements. The amount of total protein was determined by BCA protein assay kit.

### Mucin mRNA analysis by Real-time PCR

Total RNA was extracted from colonic samples using Trizol reagent (15596–026, Invitrogen, USA) according to the operating manual. Total RNA (2 μg) was retrotranscribed into cDNA using the GoScript^™^ reverse transcription system (Promega, Shanghai, China). The reaction condition was as follows: 70 °C incubation for 10 min, then at 42 °C for 60 min, and terminated at 75 °C for 15 min. And 5 μl cDNA was used in a Real-time PCR reaction which was performed with ABI 7500 Real-time PCR system (Life technology). Gene expression was detected using SYBR Green [FastStart Universal SYBR Green Master (Rox), Roche] based PCR with GAPDH as the internal reference. The primer sequences were designed by Invitrogen Biotechnology (Shanghai) Co. LTD. (Shanghai, China) and listed in Table 2. The cycle procedures were as follows: 95 °C initial denaturation for 2 min, then 45 cycles of 95 °C denaturation for 30 s, 60 °C annealing for 30 s and 72 °C extension for 30 s. A melting curve analysis, from 55 to 98 °C, was conducted after the finish of the amplification cycles. Finally, the relative expression levels were normalized to GAPDH and calculated using the 2^-ΔΔCt^ method.

**Table 2:** Primers for Real-time PCR.

Gene	Sequences (5'-3')	ProductSize (bp)	Genbank ID
GAPDH	F: ATTGTCAGCAATGCATCCTG	102	NM-001289726.1
	R: ATGGACTGTGGTCATGAGCC		
*Mucin-1*	F: CCAAGCGTAGCCCCTATGAG	114	NM-013605.2
	R: GTGGGGTGACTTGCTCCTAC		
*Mucin-2*	F: TTCCAACCCTCCTCCTACCAC	189	NM-023566.3
	R: CTCCACCATTCCACCAGACG		

The sequences are available through GenBank (http://www.ncbi.nlm.nih.gov/nuccore/) under the accession numbers listed above.

### Western blot analysis

Total protein in the colon tissues was homogenized in ice-cold RIPA lysis buffer containing 50 mM Tris (pH 7.4), 150 mM NaCl, 1% sodium deoxycholate, 0.1% SDS, 10 mM NaF, 5 mM EDTA, 1 mM Na_3_VO_4_, and 1% protease inhibitor (Roche) for 1 min, incubated for 30 min on ice and centrifuged at 14000 g for 10 min. Protein concentration of liquid supernatant was determined using BCA assay kit (Thermo Scientific Pierce). The proteins were denatured, loaded on polyacrylamide gel (10 μg/well), separated by electrophoresis, and then transferred onto the PVDF membrane. This membrane was blocked with 5% defatted milk in TBST for 1 h and incubated at 4 °C overnight with primary antibodies against β-actin (internal control, Cell Signaling Technology), Bax, Bcl-2, ZO-1, ZO-2, claudin-1, or occluding (Santa Cruz). Subsequently, the membranes were incubated with HRP-conjugated secondary antibodies (Cell Signaling Technology), and blots were developed using the ECL detection reagents (Millipore) and analyzed by Image J software (Rawak Software, Inc. Germany).

### Statistical analysis

Data were presented as means ± standard error (SEM) and imaged using Graphpad Prism5 software (GraphPad Prism, USA). Statistical significance between groups was evaluated by one-way ANOVA followed by the Dunnett’t test. Difference was considered significant at *P* < 0.05.

## Results

### BB mitigated the clinical symptoms in DSS-induced colitis mice

**Fig 2:**
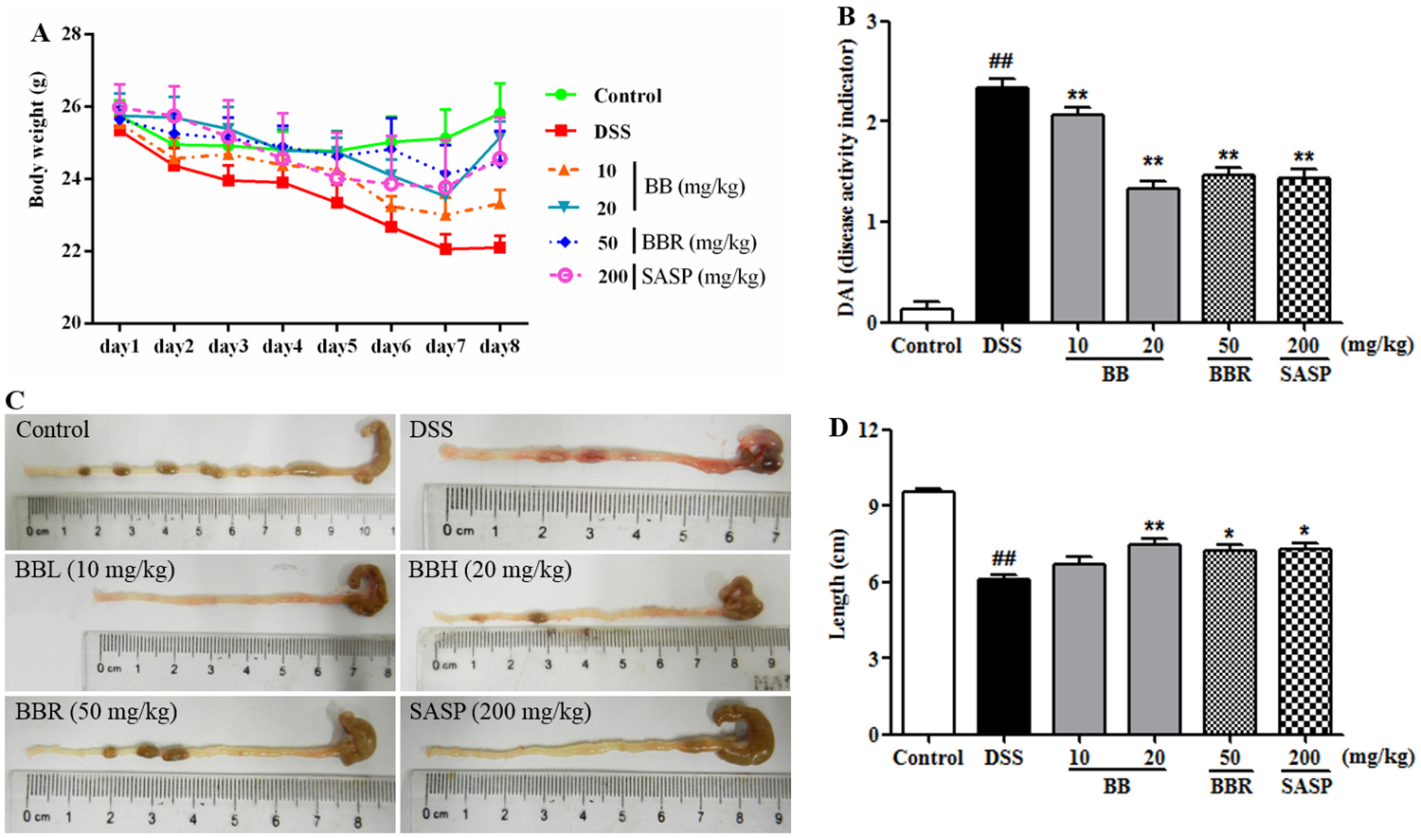
BB remitted the clinical symptoms in mice with DSS-induced colitis. (A) Bodyweight was shown as a mean value of daily weight from days 1 to 8. (B) DAI scores. (C) Gross morphology of colonic tissues. (D) Measuring result of gut length. Results are expressed as means ± SEM (n = 10). ^##^*P <* 0.01 vs. Control group,*P < 0.05 and**P < 0.01 vs. DSS group.

Consistent with previous study [31], challenging mice with DSS administration induced acute colitis characterized by bloody diarrhea, ulceration, colon shortening and loss of body weight, indicative of successful establishment of the UC murine model with typical symptoms. As shown in Fig 2A, the bodyweight of mice in control group was gradually increased, while the bodyweight of DSS group was sustainably and substantially reduced compared to the control group. In contrast, the bodyweight of mice in BB (20 mg/kg), BBR (50 mg/kg) or SASP (200 mg/kg) groups recovered greatly from day 5 to day 8.

The DAI score, which is an indicator of the severity of colitis, is based on the results including weight loss, stool charter and fecal occult blood [32]. As shown in Fig 2B, DAI score for the DSS group was significantly higher as compared to the control group (*P <* 0.01). However, the DAI scores for the groups treated with BB (20 mg/kg), BBR or SASP were all significantly lower as compared to the DSS group (*P <* 0.05). As expected, colitis manifested severe intestinal inflammation, leading to colon shortening [5], a morphological indicator of colon inflammation. In accordance with the DAI, the colon of mice in DSS group was distinctly shorter than that of the control group on day 8, while mice treated with BB, BBR or SASP exhibited remarkably ameliorating colon shortening compared to the DSS-treated model mice (Fig 1C and 1D).

### BB attenuated the colonic histopathological changes in mice colitis

**Fig 3:**
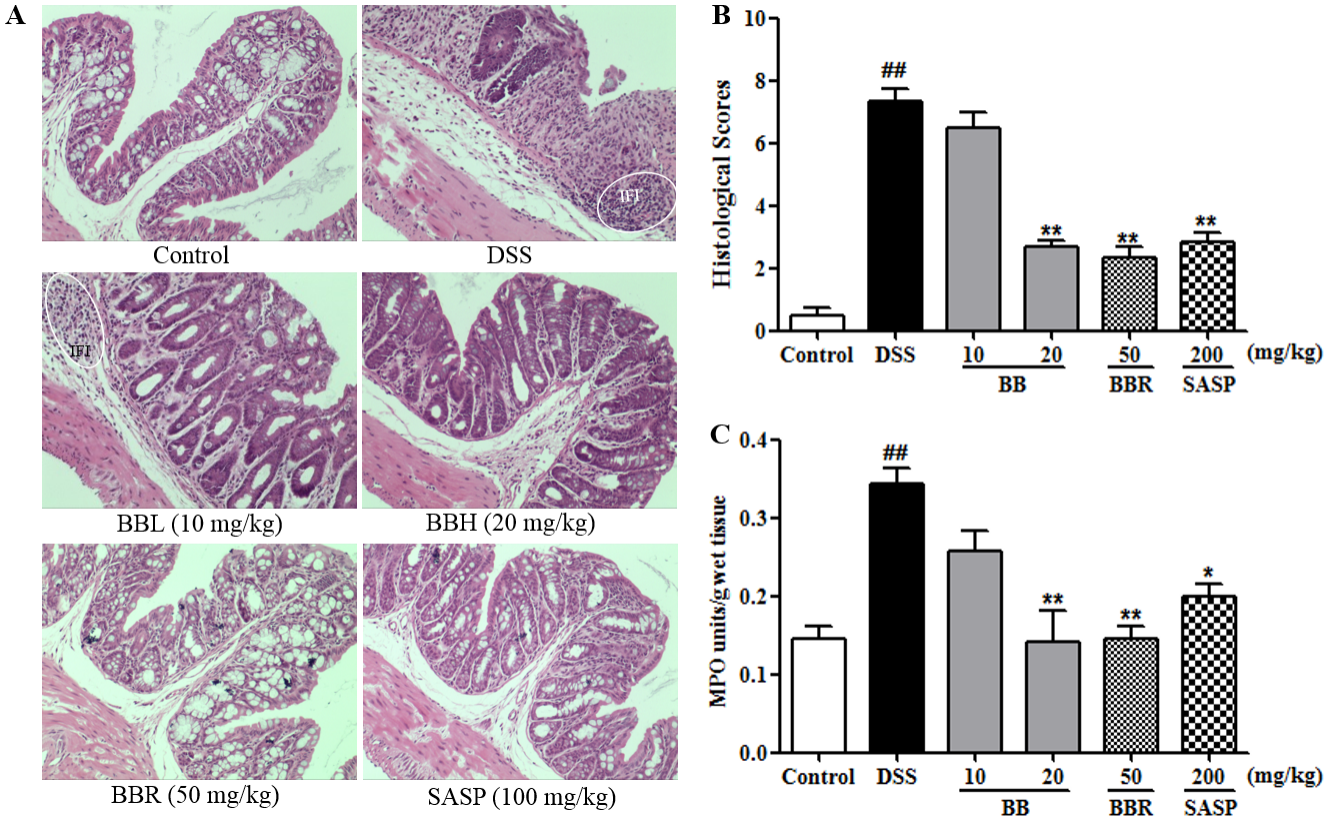
BB mitigated the deteriorating colonic histopathological changes in mice with DSS-induced colitis. (A) Representative H&E staining images of colonic sections from different treatments. (B) Histopathology scores. (C) MPO activity. The results shown are means ± SEM (n = 6). ^##^*P <* 0.01 vs. Control group, *P < 0.05 and **P < 0.01vs. DSS group.

Histological characteristics of the colons were subsequently examined by H&E staining. Mice in control group maintained integrated normal colonic structures (Fig 2A), characteristic of clear outer membrane, muscular layer, submucosa and mucosa. However, DSS treatment elicited deteriorating pathological alternations, such as epithelium cell layer demolition, crypt lack, mucosa inflammation and cell infiltration, resulting in significantly higher histological scores as compared with the control group (Fig 3B). This colonic damage as determined pathologically was paralleled to that of macroscopically visible colonic injures. By contrast, mice treated with BB (20 mg/kg), BBR (50 mg/kg) or SASP (200 mg/kg) showed remarkably ameliorated intestinal injury, relieved mucosa inflammation and significantly decreased histological scores (Fig 3B) as compared to the DSS mice. Treatment with BB (10 mg/kg) was shown to only slightly ease the gut structural lesions, however, the histological score difference failed to reach statistical significance when compared with the DSS group.

MPO activity, an index to evaluate the neutrophil infiltration and inflammation, is believed to be proportional to the concentration of neutrophils in lesion tissue [33]. As shown in Fig 3C, DSS treatment significantly increased the MPO activity as compared with the control (*P* < 0.01). Whereas, BB (20 mg/kg), BBR (50 mg/kg) or SASP (200 mg/kg) treatment was found to appreciably decrease the elevated MPO activity induced by DSS, which was congruent with the histological findings.

### BB decreased colonic cytokines concentration in mice with colitis

**Fig 4:**
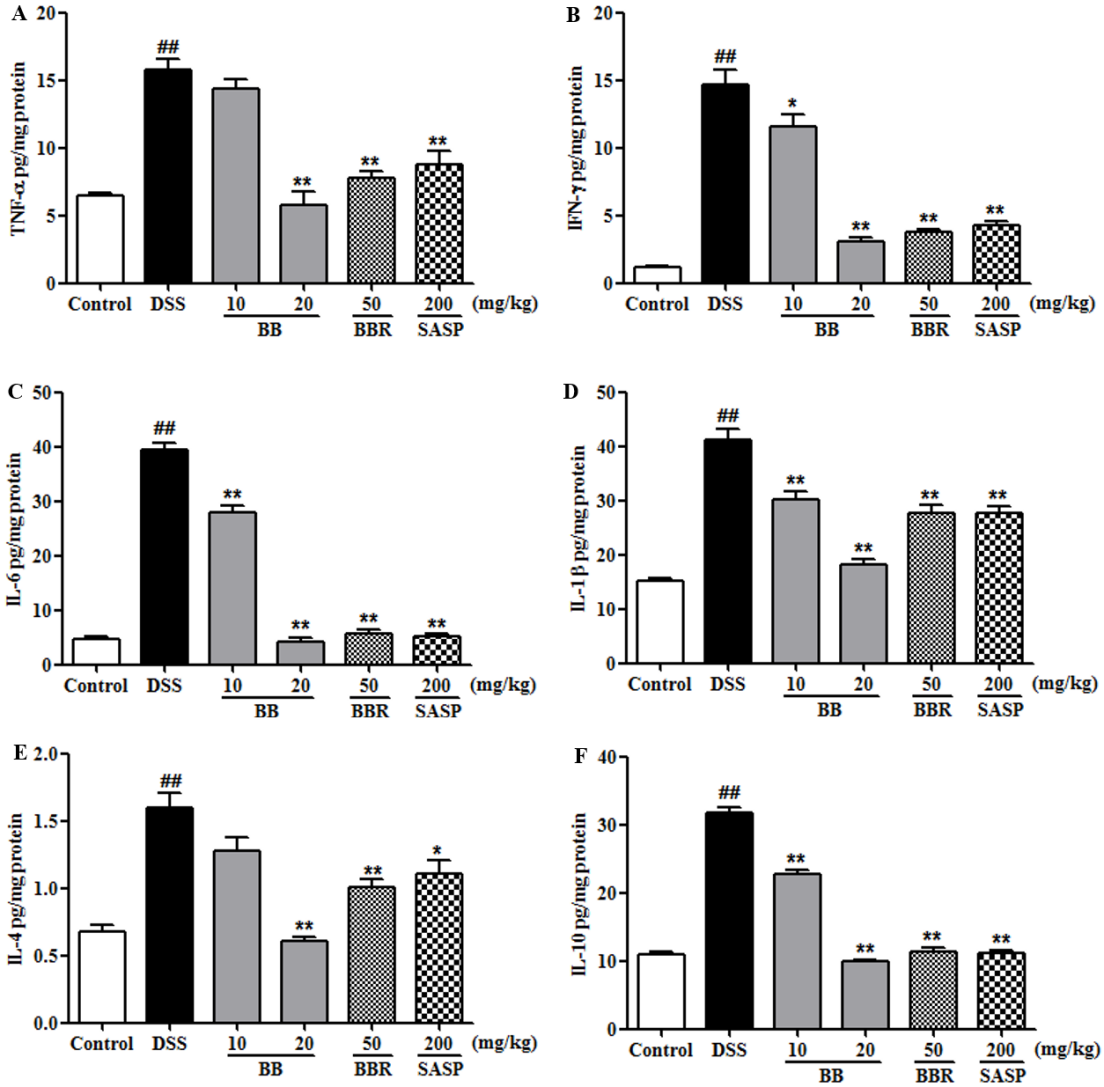
BB suppressed colonic levels of inflammatory cytokines in mice with DSS-induced colitis. (A) TNF-α, (B) IFN-γ, (C) IL-6, (D) IL-1β, (E) IL-4, and (F) IL-10 levels were examined by ELISA. The results shown are means ± SEM (n = 10). ^*##*^*P <* 0.01 vs. Control group, *P < 0.05 and **P < 0.01 vs. DSS group.

Development of colitis is often associated with chronic inflammation, characterized by inflammatory cellular infiltration and series of cytokines secretion, such as TNF-α, IFN-γ, IL-6, IL-1β, IL-4, and IL-10 [31, 34]. Cytokines perform essential roles in the intestinal immune system and are shown to have a central role in the pathophysiology of UC. Hence, we detected these above-mentioned indicators to estimate the potential anti-inflammatory effect of BB on DSS-induced colitis. Results indicated that DSS treatment induced remarkable augments of colonic levels of cytokines (TNF-α, IFN-γ, IL-6, IL-1β, IL-4 and IL-10) compared to the control group (Fig 4). However, treatment with BB (especially at the dose of 20 mg/kg), BBR (50 mg/kg) or SASP (200 mg/kg) obviously reversed these elevations induced by DSS (*P* < 0.05). These data indicated that treatment with BB effectively ameliorated the colonic inflammation caused by DSS.

### BB preserved intestinal barrier function in the colon of DSS-treated mice

**Fig 5:**
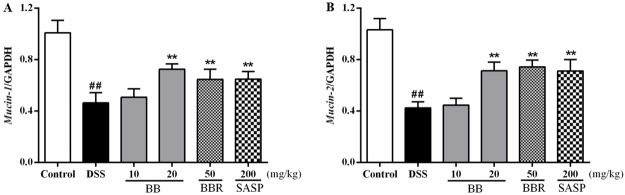
BB prevented disruption of intestinal integrity in mice with DSS-induced colitis. Gene expression was normalized to GAPDH in each group. The results shown are means ± SEM (n = 10). ^##^ *P <* 0.01 vs. Control group, *P < 0.05 and **P < 0.01 vs. DSS group.

The colonic epithelium is composed of trefoil peptides such as mucin-1 and mucin-2, which play an important role in maintaining the barrier function of colon. To evaluate the potential effect of BB on colonic mucins, mRNA levels of the mucins (*mucin-1* and *mucin-2*) in the colon of DSS-colitis mice were measured by RT-PCR. As shown in Fig 5, the gene expression of *mucin-1* and *mucin-2* was observably suppressed by DSS treatment relative to the control (*P* < 0.01). On the contrary, BB (20 mg/kg), BBR (50 mg/kg) and SASP (200 mg/kg) were shown to significantly upregulate the expression of these two genes (*P* < 0.01).

Intestinal epithelial tight junctions play a key role in protecting against inflammation, and disrupted tight junctions are a main cause of intestinal barrier dysfunction and inflammation. Apoptosis is also a well-known inducer of mucosal injury in many intestinal diseases, which can result in barrier dysfunction. Therefore, maintaining TJs and suppressing apoptosis to protect barrier function may provide benefits in the therapy of UC. To verify the involvement of apoptosis and epithelial TJ, the expression levels of apoptosis-associated proteins (Bax, Bcl-2) and TJ-associated proteins (ZO-1, ZO-2, claudin-1, occluding) were determined on colon tissues by Western blotting.

**Fig 6:**
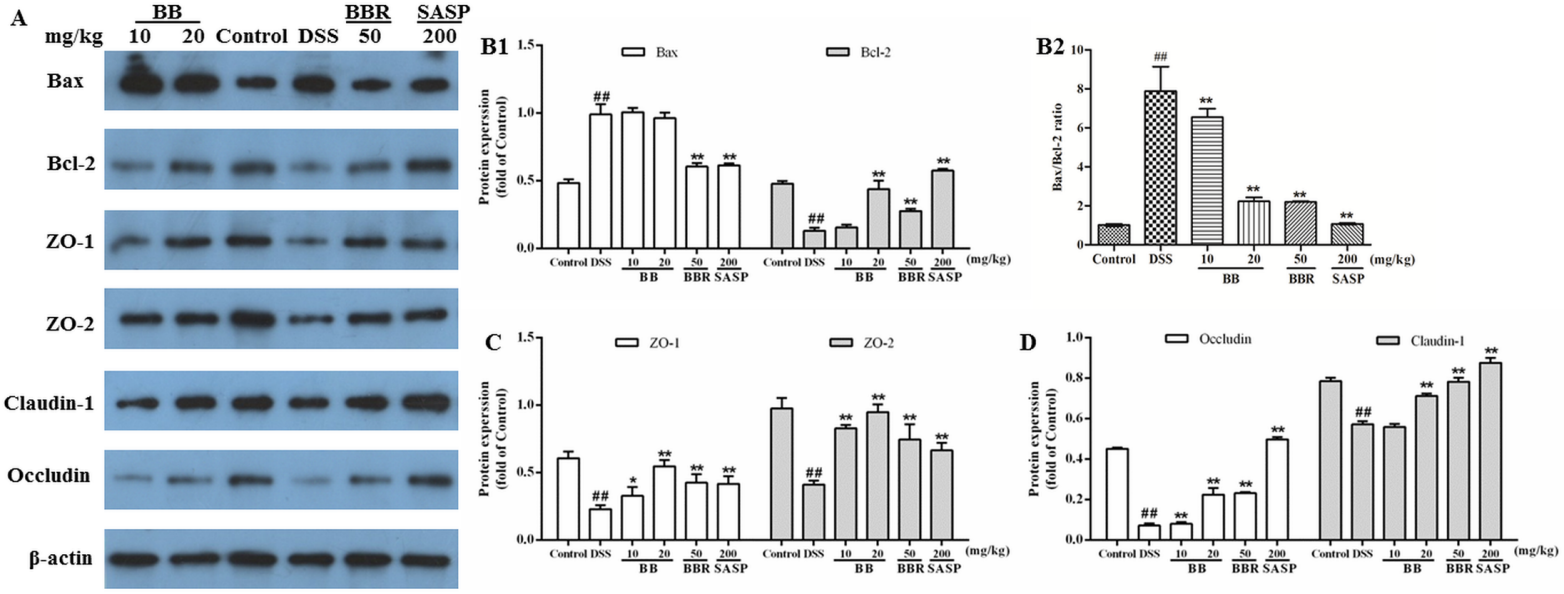
BB alleviated the apoptosis and recovered the intestinal barrier function in mice with DSS-induced colitis. Homogenates of colonic tissue were analyzed by Western blotting and representative images were shown (A). Quantitative results of expression levels of apoptosis-related proteins Bax and Bcl-2 (B1), Bax/Bcl-2 expression ratio (B2), tight junction-associated proteins (ZO-1 and ZO-2) (C), and occludin and claudin-1 (D). β-actin was used as a loading control. The results shown are means ± SEM (n = 6). ##*P* < 0.01 vs. Control group, *P < 0.05 and **P < 0.01 vs. DSS group.

As shown in Fig 6A, the down-regulation of anti-apoptotic Bcl-2 and up-regulation of pre-apoptotic Bax were observed during DSS-induced colitis. By contrast, BB treatment markedly augmented the expression of Bcl-2 (*P <* 0.01) and slightly attenuated the expression of Bax, resulting in significantly decreased Bax/Bcl-2 ratio (*P <* 0.01) in colonic tissues. Furthermore, the protein expression of ZO-1, ZO-2, claudin-1 and occluding was all significantly down-regulated in mice treated with DSS alone, as compared with normal mice (*P <* 0.01). However, these proteins expression was prominently up-regulated by BB (especially at 20 mg/kg), BBR (50 mg/kg) and SASP (200 mg/kg) (Fig 6B and 6C). These results indicated that BB might prevent DSS-induced disruption of intestinal integrity by preserving tight junctional function and decreasing apoptosis.

## Discussion

Berberine (BBR) is a commonly-used therapeutic agent for the treatment of gastroenteritis and infectious diarrhea in China. Previous study has suggested that BBR remitted DSS-induced colitis by inhibiting inflammatory responses in colonic macrophages and epithelial cells in DSS-treated mice [14]. Even though BBR possesses a low oral bioavailability, it has exhibited appreciable biological activities *in vivo* and the levels of its major metabolites like BB was relatively high [18]. These reports suggested that the metabolites might be active contributors to the biological activities of BBR *in vivo*. Indeed, various studies have revealed that the metabolites of BBR showed similar bioactivities. BB, the main primary metabolite of BBR, was more lipophilic and could be absorbed more efficiently in intestine than BBR [19]. Furthermore, BB was rapidly absorbed and widely distributed in various tissues [35]. Therefore, we hypothesized that BB might have similar beneficial or superior effect to BBR on UC. In the present work, pioneering endeavor was initiated to investigate the anti-UC effect of BB on DSS-induced colitis in comparison with BBR, and probe the possible underlying mechanism.

DSS-induced experimental colitis, one of the most widely used models of chemically induced colitis, has clinical symptoms similar to human UC [36–38]. In the present work, treatment with BB, as well as BBR, significantly ameliorated clinical symptoms of UC such as diarrhea, stool charter, bloody stool and bodyweight loss, thereby reducing the DAI score. Additionally, administration with BB (20 mg/kg) relieved signs of inflammation and mucosal injury as indicated by improved colon length and higher histological score, suggesting a protective effect of BB in the model of colitis. Noteworthy, BB was shown to exert similar effect to BBR and positive control SASP in attenuating DSS-induced UC with much smaller dosage.

Inflammatory responses, characteristic of gut barrier disruption in IBD, play a vital role in the pathogenesis of UC [39, 40]. Gut inflammation activates accumulated colonic macrophages, resulting in over-production of pro-inflammatory cytokines, such as TNF-α, IL-1β, IL-6 and IL-4, which further deteriorate UC [41] [42]. IFN-γ released by excessive function of Th1 cells is generally associated with intestinal inflammation especially in IBD [43, 44]. Furthermore, the level of MPO activity, proportional to the concentration of neutrophils in lesion tissue, was a common index to evaluate the neutrophil infiltration and inflammation [45]. Preceding investigations have reported BBR alleviated experimental colitis induced by DSS or trinitrobenzene sulfonic acid in rodent, which was closely associated with its anti-inflammatory activity. In this study, we found the induced productions of TNF-α, IFN-γ, IL-6, IL-4 and IL-1β by DSS treatment were dramatically suppressed by BB (especially at the dose of 20 mg/kg), BBR and SASP (Fig 4A–4E, *P <* 0.05). Besides, BB and BBR also decreased the levels of IL-10 in DSS-treated colitis mice (Fig 4F, *P <* 0.05). IL-10, which was mainly secreted by Treg cells, has been defined as anti-inflammatory cytokine. However, report has also suggested that T cells in UC patients expressed high level of IL-10 in intestine [46, 47], and high-level of IL-10 may result in the induction of pro-inflammatory cytokines in IBD [47]. The increased IL-10 in DSS mice in the present work was consistent with our preceding investigation [48] and other previous reports [49]. These results showed that suppression of inflammatory response in the colonic tissue of mice might contribute to the ameliorative effect of BB against DSS-induced colitis, which paralleled the histological evidence of protection.

The barrier dysfunction involved in the initiation and propagation of inflammation, and inflammatory cytokines could induce barrier dysfunction with vicious cycles. Most pro-inflammatory cytokines (e.g. IFN-γ, TNF-α, and IL-1β) could reduce key TJ-associated proteins such as intracytoplasmic proteins (ZO-1, ZO-2) and transmembrane proteins (occluding and claudin), resulting in disruption of intestinal TJ barrier and the development of intestinal inflammation [50]. Furthermore, the integrity of intestinal structure critically involves the function of the mucous layer, which comprised of mucin (primarily including mucin-1 and mucin-2) excreted by enterocyte, exerting lubricant and protective effect on intestinal tract [7]. Therefore, restoring intestinal barrier function may be a potentially beneficial strategy in the treatment of UC. Previous studies have indicated that BBR promoted barrier function in human retinal pigment epithelial cells, endothelial cells and Caco-2 human epithelial colorectal adenocarcinoma cells [51]. In this study, we observed the loss of colonic TJ (ZO-1, ZO-2, occludin and claudin-1) and colonic mucin (mucin-1 and mucin-2) expression in DSS-induced mice were dramatically suppressed by BB and BBR (Fig 6, *P <* 0.05). These results indicated that the ameliorative effect of BB against DSS-induced colitis might be tightly associated with its protection on intestinal mucosal barrier via enhancing the expression of TJ proteins and mucins.

Besides, increased apoptosis in intestinal epithelial cells also does serious damage to the mucosal barrier and induces gut inflammation. This has been reported in patients with UC, which suggested that the loss of epithelial cells in active UC was induced mainly by apoptosis in crypts of involved and adjacent uninvolved areas [52]. The same phenomenon also has been observed in CD patients [53] and murine models of colitis [54]. The key of keeping normal tissue homeostasis is to achieve balance of epithelial cell apoptosis and proliferation. The present study suggested that BB treatment significantly up-regulated the anti-apoptotic Bcl-2 protein expression and markedly decreased the Bax/Bcl-2 ratio, indicating the anti-apoptosis effect might contribute to the protection of BB against DSS-induced experimental UC.

In the current work, BB, the demethylation metabolite of BBR, was found to be more pharmacologically active than BBR in alleviating the DSS-induced colitis in mice, which was in concert with previous reports that BB possessed better hepatoprotective property [55] and OH scavenging activity [56] than BBR. These results suggested that the metabolite BB might contribute to the biological activities of BBR *in vivo*. The BB molecule is very similar to BBR except that the methoxy (OCH_3_) group at position 9 is replaced byhydroxyl group (OH) group, resulting in enhanced anti-UC activity. Indeed, the OH group at the C-9 position of BB was believed to be essential for its biological activities [56, 57]. Although the pharmacokinetic and pharmacodynamic relationships between BB and BBR *in vivo* merited further in-depth investigation, this observation provided important implication for the design of novel therapeutic entity with improved anti-UC efficacy. Furthermore, BB is also a naturally-occurring protoberberine alkaloid oriented from the medicinal plant *Berberis vulgaris*, which has been shown to possess anti-UC effect. Hence, it was deduced that BB might be one of the active principle obligatory for biological effect of *Berberis vulgaris* in the treatment of UC.

Since BB was a main metabolite of BBR, which serves as a safe and effective agent for the treatment of gastrointestinal diseases in clinic, BB was expected to provide a wide margin of safety. Furthermore, BB possessed similar anti-UC effect to BBR and the positive control SASP with much smaller dosage. Hence, BB might have the potential to be further developed into an effective candidate for the treatment of UC. Future studies for elucidating the detailed molecular mechanism underlying the anti-UC effect of BB, long-term safety and pharmacokinetics would likely pave an avenue for promising drug development process.

## Conclusion

This was the first report to indicate that BB had pronounced protective effect on DSS-induced colitis by significantly easing the clinical symptoms, suppressing the inflammatory response and repairing mucosal barrier function. These effects were mediated, at least in part, via amelioration of pro-inflammatory cytokines production and modulation of TJ proteins, mucins genes and apoptosis-related proteins in the colonic tissue. The anti-UC effect of BB was similar to that of BBR and positive control SASP with much lower dosage. Therefore, we speculated that BB might be one of the active metabolites of BBR responsible for its anti-UC effect, and BBR together with BB might form the material basis of BBR *in vivo*. BB was believed to have the potential to be further developed into a promising therapeutic agent in the treatment of UC.
